# A case report of a novel *HIST1H1E* mutation and a review of the bibliography to evaluate the genotype–phenotype correlations

**DOI:** 10.1002/mgg3.2273

**Published:** 2023-08-21

**Authors:** Wenjing Zhao, Yinhong Zhang, Tao Lv, Jing He, Baosheng Zhu

**Affiliations:** ^1^ Department of Medical Genetics, First People's Hospital of Yunnan Province Kunming China; ^2^ Affiliated Hospital of Kunming University of Science and Technology Kunming China; ^3^ National Health Commission Key Laboratory of Preconception Health Birth in Western China Kunming China

**Keywords:** de novo variant, genotype–phenotype correlation, *HIST1H1E*, neurodevelopmental disorders, Rahman syndrome

## Abstract

**Background:**

*HIST1H1E* is a member of the H1 gene family. Excess de novo likely gene‐disruptive variants involving the C‐terminal tail of *HIST1H1E* have been reported in neurodevelopmental disorders. Although clinical phenotypes in some patients have been described in single studies, few studies have reviewed the genotype and phenotype relationships using a relatively large cohort of patients with *HIST1H1E* variants.

**Methods:**

Whole‐exome sequencing (WES) was performed on the proband. The variant was validated using Sanger sequencing in both proband and parents. Published *HIST1H1E* variants in neuropsychiatric disorders were reviewed.

**Results:**

Herein, we reported a new de novo frameshift mutation in *HIST1H1E* (NM_005321.2, c.416_419dupAGAA, p.Ala141GlufsTer56) in an individual with Rahman syndrome. To explore the genotype–phenotype correlations for *HIST1H1E* variants in neurodevelopmental disorders, we comprehensively curated and summarized 23 variants and the clinical features from 52 patients. Our findings revealed that likely gene‐disrupting variants in *HIST1H1E* contribute to a wide range of neurodevelopmental phenotypes. We observed the common phenotypes including craniofacial features, ID, hypotonia, and autism/behavior problem in patients with *HIST1H1E* variants. While the different genotypes corresponding to different phenotypes or the same phenotype were also observed.

**Conclusion:**

These data provide scientific evidence for the genetic diagnosis and precision clinical management.

## Graphical abstract

We reported a new de novo frameshift mutation in HIST1H1E (NM_005321.2, c.416_419dupAGAA, p.Ala141GlufsTer56) in an individual with Rahman syndrome. we comprehensively curated and summarized 23 variants and the clinical features from 52 patients. Our findings revealed that likely gene‐disrupting variants in HIST1H1E contribute to a wide range of neurodevelopmental phenotypes.

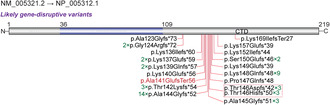

## 1 INTRODUCTION

*HIST1H1E* (MIM:142220), encoded by *HIST1H1E*, is a globular domain‐containing factor. *HIST1H1E* binds to linker DNA between nucleosomes forming the chromatin fiber to modulate gene expression and DNA replication, recombination, and repair (Bayona‐Feliu et al., 2017; Fan et al., 2005; Happel et al., 2009; Hendzel et al., 2004; Hergeth & Schneider, 2015; Izzo et al., 2013). With the rapid development of next‐generation sequencing technologies, *HIST1H1E*, a member of the H1 gene family, was first reported as a causative gene of schizophrenia (Xu et al., 2012). Variants within *HIST1H1E* have been frequently reported in neurodevelopmental disorders characterized by mild to severe intellectual disability (ID), overgrowth which may appear in early infancy but become progressively closer to average in adults, and facial deformity, such as high hairline, prominent forehead, bitemporal narrowing, downward slant palpebral fissures, deepset eyes, hypertelorism, and so on, indicating a complex phenotypic outcome. Despite the advent of next‐generation sequencing has accelerated to elucidate the genetic causes of neurodevelopmental disorders, many individuals with neurodevelopmental disorders remain without a genetic diagnosis because of the fuzzy correlations between genotype and phenotype. Importantly the GeneReviews, as they already show data from 47 cases, which is critical in the understanding of genotype–phenotype relationships. Here, we report a new de novo frameshift mutation within the *HIST1H1E* gene in a patient with Rahman syndrome. In addition, we comprehensively curated 23 variants from 52 individuals with neurodevelopmental disorders from the published literature and online databases and this study to investigate the genotype and phenotype relationships. We thoroughly evaluate more detailed phenotypic aspects of all published cases harboring *HIST1H1E* mutations. The phenotypic similarities and diversity between different types of variants were analyzed and discussed. The results will be beneficial for the genetic diagnosis and clinical management of patients with *HIST1H1E* variants, and promote the selection of treatment targets in the future.

## 2 METHODS

### 2.1 Subjects, mutation identification, and validation

Peripheral blood samples and clinical data from the subjects were collected with informed consent. Genomic DNA was extracted from the whole blood using a standard proteinase K digestion and phenol–chloroform method. Whole‐exome sequencing (WES) was performed as previously described (Mamanova et al., 2010). Variants were annotated with ANNOVAR (2019Oct24) (Wang et al., 2010). The candidate *HIST1H1E* (GenBank accession number: NM_005321.2) variant was validated by Sanger sequencing. Primers (5′‐TGGCTATGACGTGGAGAAGAAC‐3′ and 5′‐GGTTTAACTGCTTTGGCCTTC‐3′) were designed using Primer3 software and the UCSC reference genome (GRCh37/hg19). We have submitted the variants to ClinVar (https://www.ncbi.nlm.nih.gov/clinvar/).

### 2.2 Review of the *HIST1H1E* genotype and phenotype data

To comprehensively identify previously published patients with *HIST1H1E* variants, we checked Clinvar and Human Gene Mutation Database (Online Professional Version) to identify any potential *HIST1H1E* pathogenic variants. We then carefully curated the previous individual case reports as well as large‐scale cohort studies. The curated variants were subsequently classified following the standards and guidelines for the interpretation of sequence variants from the American College of Medical Genetics and Genomics (ACMG) (Richards et al., 2015). The detailed molecular and phenotypic information was curated from corresponding studies if available (Table S1).

## 3 RESULTS

### 3.1 A new *HIST1H1E* frameshift mutation and the clinical characterizations

**FIGURE 1:**
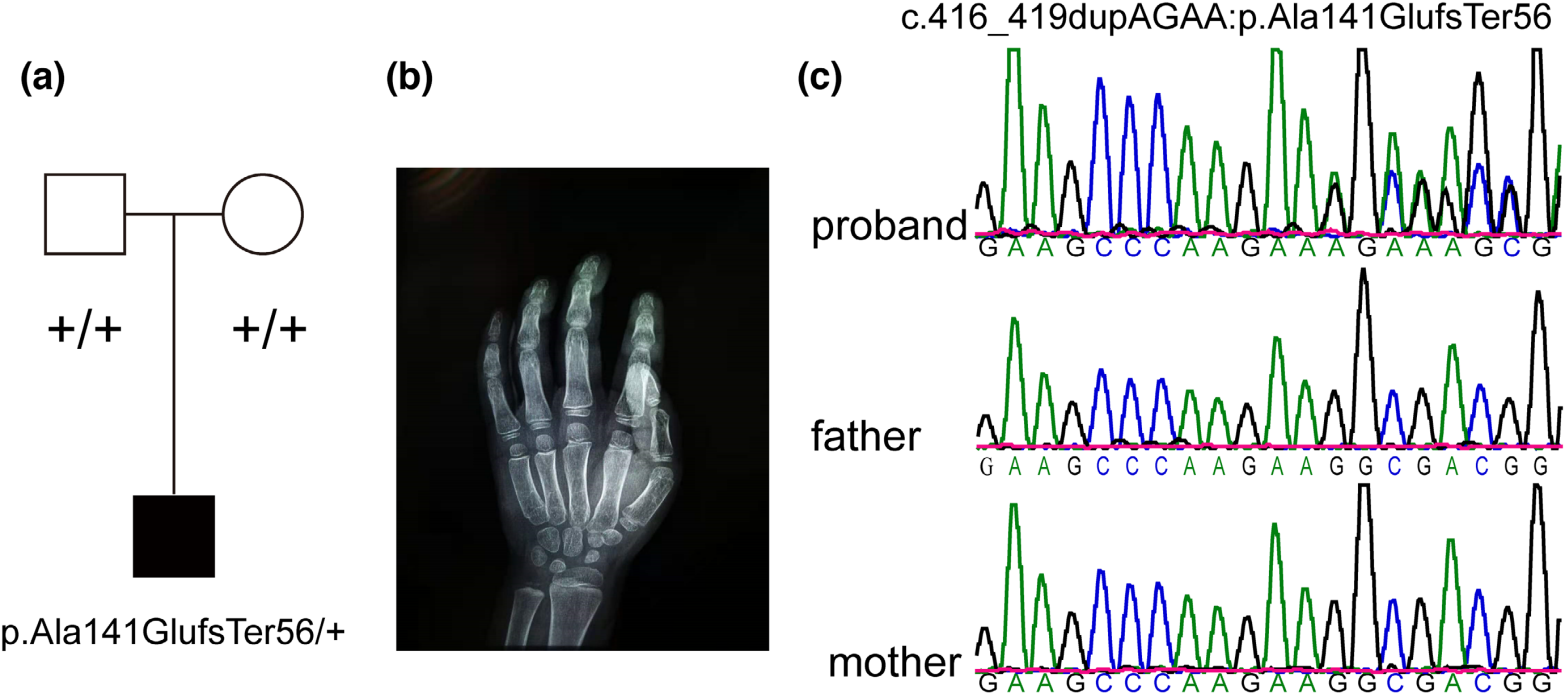
A new de novo frameshift variant in *HIST1H1E* (NM_005321.2, c.416_419dupAGAA, p.Ala141GlufsTer56) in an individual with Rahman syndrome. (a) The segregation of this variant in the family; (b) A hand radiograph; (c) Sanger sequencing results.

The male proband is the first child of his healthy, nonconsanguineous Chinese parents. He was born prematurely at 33 weeks with the weight of 2.55 kg and height of 44 cm. In the neonatal period, he cried rarely and had pneumonia. No asphyxia nor jaundice. On examination at 4 years of age, his weight was 24 kg (+3 SD), height 113 cm (+2 SD), and head circumference 52.5 cm (+1 SD). Developmental milestones were delayed. He was able to crawl at the age of 2.5 years. He was able to sit without support at the age of 1 year. He was able to walk without support at the age of 3 years. Speech development was more delayed. After the age of 2 years, he started to speak his first phrases. At the age of 4 years, he could only speak a few words. His behavior was characterized by stereotypic movements and sleeping problems. Developmental regression was reported. He presents a severe ID and autism. Brain MRI performed at the age of 5 months showed mild bilateral ventricular enlargement, slightly wide cavum septum pellucidum, the left temporal extracerebral space slightly wider, thin corpus callosum, delayed myelination, and pituitary gland with nodular protrusion of superior margin. A moderate hearing deficit was noted in only the left ear. He has no hypotonia or scoliosis. Craniofacial features characterized by a high hairline, prominent forehead, bitemporal narrowing, epicanthus, telecanthus, hypertelorism, a low and broad nasal bridge, upturned and full nasal tip, short philtrum, and low set ears. His hair is sparse and delayed growth at the temples. Abnormal dentition including widely spaced teeth, pointed teeth, and dental caries was presented. Other physical features included myopia, astigmatism, flat feet, simple syndactyly of the toes, thin nails, multiple nevi, atrial septum defect, bilateral cryptorchidism, and hypothyroidism. He had advanced bone age (Figure 1b). Medical concerns included hypothyroidism. G‐banded karyotyping revealed a normal karyotype (46, XY) for the proband. WES identified a de novo frameshift mutation in *HIST1H1E* (NM_005321.2, c.416_419dupAGAA:p.Ala141GlufsTer56) (Figure 1b). Sanger sequencing confirmed this variant in the proband but not in the parents (Figure 1a,c).

### 3.2 Mutation pattern and genotype–phenotype correlations for *HIST1H1E*

**FIGURE 2:**
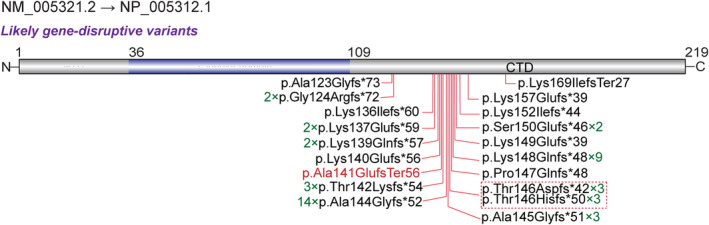
Protein model of *HIST1H1E* with all reported variants indicated. The novel de novo frameshift variant identified in this study is marked with red color. × arabic numerals represent the number of independent cases indicated in the present study.

A total of 23 frameshift variants from 52 patients (Burkardt et al., 2019; Ciolfi et al., 2020; Duffney et al., 2018; Flex et al., 2019; Helsmoortel et al., 2015; Indugula et al., 2022; Takenouchi et al., 2018; Tatton‐Brown et al., 2017; Zhao et al., 2022) with neurodevelopmental disorders were curated from the large‐scale genome‐wide sequencing studies or individual patient reports or online databases and this study (Table 1; Figure 2). All are resulting in almost identical shorter proteins that contained a shared divergent C‐terminal tail (Burkardt & Tatton‐Brown, 2020; Flex et al., 2019). In addition, there are eight recurrent nonsense variant loci (Gly124, Lys139, Thr142, Ala144, Ala145, Thr146, Thr146, Lys148). All changes were short out‐of‐frame indels and present in the carboxy‐terminal domain. None of the frameshifts had been reported in the Genome Aggregation Database (gnomAD) (Lek et al., 2016), all were classified as pathogenic or likely pathogenic according to the ACMG guidelines (Table 1).

**TABLE 1:** *HIST1H1E* variants in neuropsychiatric disorders.

Variant index	Alteration (phys.Location) (hg19, chr6)	NT change	AA change	Function	Domain	Case Num	Origin	gnomAD	ACMG classification
V1	26156978_26156979insA	c.360_361insA	p.Ala123Glyfs*73	Frameshift	C‐terminal tail	1	de novo		Likely pathogenic
V2	26156986dupC	c.368dupC	p.Gly124Argfs*72	Frameshift	C‐terminal tail	2	Undetermined	–	Likely pathogenic
V3	26157024_26157025insT	c.406_407insT	p.Lys136Ilefs*60	Frameshift	C‐terminal tail	1	de novo	–	Likely pathogenic
V4	26157025dupA	c.407dupA	p.Lys137Glufs*59	Frameshift	C‐terminal tail	1	de novo	–	Likely pathogenic
V5	26157026dupG	c.408dupG	p.Lys137Glufs*59	Frameshift	C‐terminal tail	1	de novo	–	Likely pathogenic
V6	26157032dupC	c.414dupC	p.Lys139Glnfs*57	Frameshift	C‐terminal tail	2	de novo (1)	–	Likely pathogenic
V7	26157034dupA	c.416dupA	p.Lys140Glufs*56	Frameshift	C‐terminal tail	1	de novo	–	Likely pathogenic
V8	26157032dupAGAA	c.416_419dupAGAA	p.Ala141GlufsTer56	Frameshift	C‐terminal tail	1	de novo	–	Likely pathogenic
V9	26157043delinsAG	c.425delinsAG	p.Thr142Lysfs*54	Frameshift	C‐terminal tail	1	Undetermined	–	Likely pathogenic
V10	26157043_26157049del insAGGGGGTT	c.425_431delinsAGGGGGTT	p.Thr142Lysfs*54	Frameshift	C‐terminal tail	2	de novo	–	Likely pathogenic
V11	26157048dupG	c.430dupG	p.Ala144Glyfs*52	Frameshift	C‐terminal tail	14	de novo (9)	–	Pathogenic
V12	26157049dupC	c.431dupC	p.Ala145Glyfs*51	Frameshift	C‐terminal tail	1	de novo	–	Likely pathogenic
V13	26157051dupG	c.433dupG	p.Ala145Glyfs*51	Frameshift	C‐terminal tail	2	de novo	–	Likely pathogenic
V14	26157053dupC	c.435dupC	p.Thr146Hisfs*50	Frameshift	C‐terminal tail	3	de novo (2)	–	Likely pathogenic
V15	26157054_26157076del23	c.436_458del23	p.Thr146Aspfs*42	Frameshift	C‐terminal tail	3	de novo (2)	–	Likely pathogenic
V16	26157055_26157056del	c.437_438del	p.Pro147Glnfs*48	Frameshift	C‐terminal tail	1	de novo	–	Likely pathogenic
V17	26157059dupC	c.441dupC	p.Lys148Glnfs*48	Frameshift	C‐terminal tail	9	de novo (7)	–	Pathogenic
V18	26157062_26157084del23	c.444_466del23	p.Lys149Glufs*39	Frameshift	C‐terminal tail	1	de novo	–	Likely pathogenic
V19	26157064 dupA	c.446dupA	p.Ser150Glufs*46	Frameshift	C‐terminal tail	1	de novo	–	Likely pathogenic
V20	26157065dupG	c.447dupG	p.Ser150Glufs*46	Frameshift	C‐terminal tail	1	de novo	–	Likely pathogenic
V21	26157072_26157073insT	c.454_455insT	p.Lys152Ilefs*44	Frameshift	C‐terminal tail	1	de novo	–	Likely pathogenic
V22	26157082dupC	c.464dupC	p.Lys157Glufs*39	Frameshift	C‐terminal tail	1	de novo	–	Likely pathogenic
V23	26157123_26157124insT	c.505_506insT	p.Lys169IlefsTer27	Frameshift	C‐terminal tail	1	de novo	–	Likely pathogenic

*Note*: *HIST1H1E* GenBank accession number (NCBI Reference Sequence): NM_005321.2.

**FIGURE 3:**
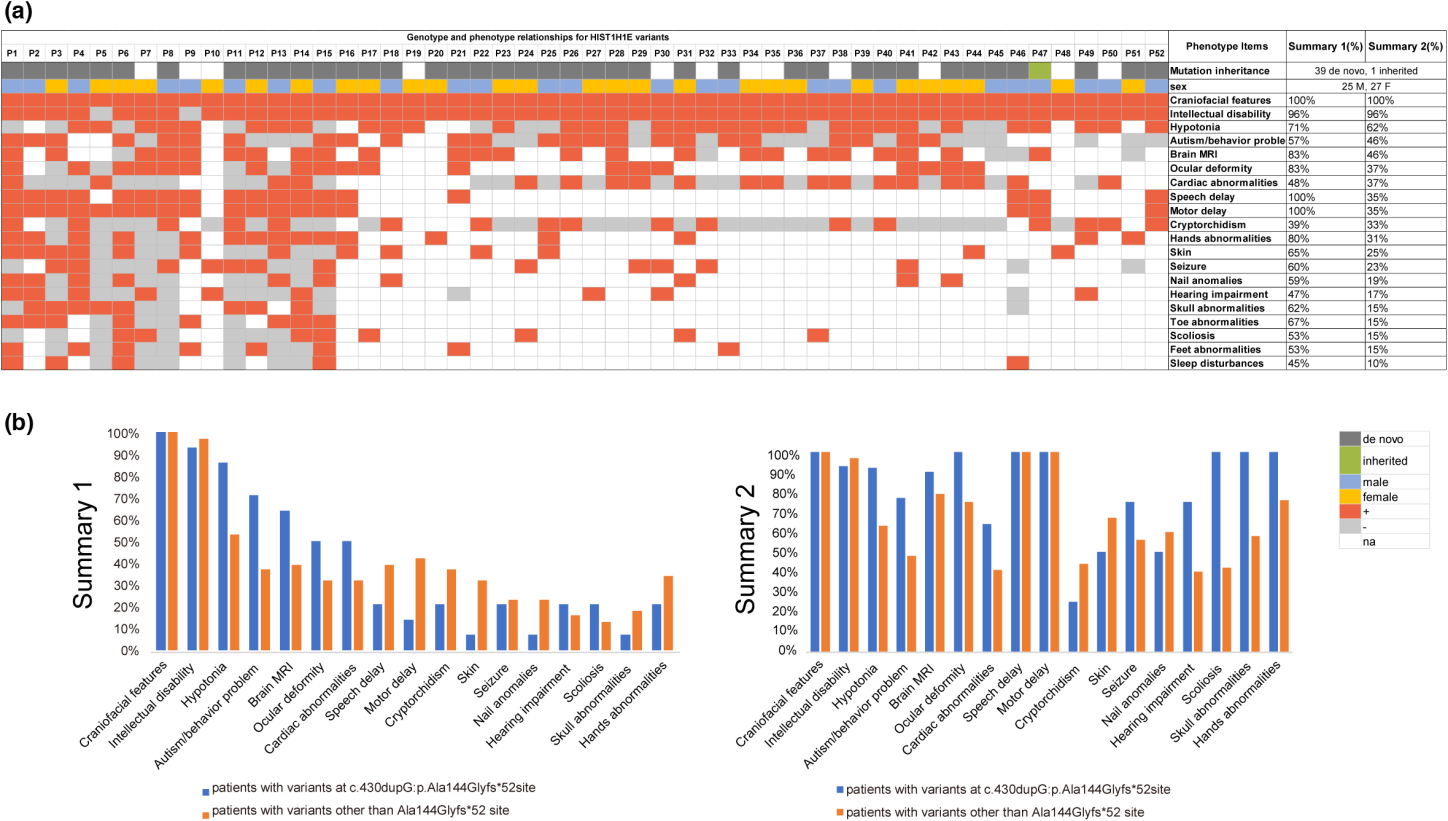
Genotype–phenotype correlations for *HIST1H1E* (GenBank accession number: NM_005321.2). (a) The key clinical presentations of patients with all reported *HIST1H1E* variants. Events in dark gray are de novo, blue are male, yellow are female, red are present, light gray are absent, and white are unreported. MRI, magnetic resonance imaging. (b) Comparison results of clinical presentations between patients with variants at c.430dupG:p.Ala144Glyfs*52 site and patients with variants other than Ala144Glyfs*52 site. The blue bars represent patients with variants at c.430dupG:p.Ala144Glyfs*52 site and the orange bars represent patients with variants other than the Ala144Glyfs*52 site. Summary 1 (%) represents the number of individuals reported to have the specific phenotype divided by the number of individuals only evaluated for this specific phenotype (“+” and “−”). Summary 2 (%) represents the number of individuals reported to have the specific phenotype divided by the number of individuals who were analyzed for genotype–phenotype correlation regardless of whether they were evaluated for this specific phenotype (“+,” “−,” and “na”).

The clinical presentations of patients with *HIST1H1E* variants were curated possibly and summarized to correlate the genotype–phenotype relationships. Finally, clinical information of the 52 patients was presented in Table S1, 25 males and 27 females. The clinical features of the 52 individuals with *HIST1H1E* variants were summarized in Figure 3a. Overall, although the phenotypes of patients are complex, several symptoms that are common to most patients are observed. 96% (50/52) of subjects were reported with variable developmental delay (DD)/ID. All subjects had distinctive craniofacial features characterized by high hairline (45/52), prominent forehead (40/52), hypertelorism (37/52), downward slant palpebral fissures (35/52), bitemporal narrowing (29/52), deepset eyes (31/52), abnormal hair (15/52), nasal bridge anomalies (13/52), epicanthus/telecanthus (12/52), full nasal tip (12/52), and low‐set and posteriorly rotated ears, etc. (12/52). Over 62% of the patients showed hypotonia (32/52), autism/behavior problem (24/52), brain MRI (24/52), ocular deformity (19/52), cardiac abnormalities (19/52), speech delay (18/52), motor delay (18/52), and hands abnormalities (16/52). Abnormal dentition including dental caries, small teeth, pointed teeth, crumbling teeth, and poorly enameled teeth was reported in 50% (26/52) patients. Cryptorchidism was reported in 68% (17/25) of boys. Other phenotypes including nail anomalies (10/52), feet abnormalities (8/52), toe abnormalities (8/52), scoliosis (8/52), and RX abnormalities (14/52), etc. were reported in Table S1.

Interestingly, all variants are located in the C terminus. The *HIST1H1E* variants escape nonsense‐mediated RNA decay since *HIST1H1E* is a single exon, intronless gene. Stable proteins have a reduced net positive charge, which disrupts the normal binding between the positively charged H1.4 linker histone and negatively charged DNA (Tatton‐Brown et al., 2017). Because the resultant abnormal stable proteins disrupt the normal proliferation rate and competence of cells, the cells hardly enter into the S phase, and undergo accelerated senescence (Flex et al., 2019). In addition, protein‐truncating variants disrupt proper compaction of DNA, which are associated with abnormally methylation of genes encoding proteins involved in synaptic transmission and neuronal function (Ciolfi et al., 2020; Flex et al., 2019).

Fourteen patients were reported with the frameshift variant (Ala144Glyfs*52). The frameshift variant at the Ala144 locus might represent a particular subtype of neurodevelopmental disorders. We attempted to compare the phenotypes between patients with Ala144 frameshift variants and other than Ala144 in order to get divergent phenotypic patterns. We found that hypotonia (12/14 vs. 20/38), autism/behavior problem (10/14 vs. 14/38), brain MRI (9/14 vs. 15/38), ocular deformity (7/14 vs. 12/38), and cardiac abnormalities (7/14 vs. 12/38) occurred with a higher prevalence in patients with Ala144 frameshift variants other than Ala144. Interestingly, patients with variants other than the Ala144 site had a higher prevalence of speech delay (15/38 vs. 3/14), motor delay (16/38 vs. 2/14), cryptorchidism (11/38 vs. 3/14), skin (12/38 vs. 1/14), nail anomalies (9/38 vs. 1/14), and skull abnormalities (7/38 vs. 1/14) than those with frameshift variants Ala144 (Figure 3a,b). Finally, we observe remarkable phenotypic differences between patients with Ala144 frameshift variants and other than Ala144 sites. This phenotype difference showed that the function of the Ala144 locus is different from that of other Ala144 locus. Further studies to explore the biological mechanisms of such frameshift variants of *HIST1H1E* are needed.

## 4 DISCUSSION

*HIST1H1E* is an essential gene located on human chromosome 6, encoding Histone H1.4 that is contained a globular domain flanked by N‐and C‐terminal tails. *HIST1H1E* is one of a family of linker histones that is responsible for higher order chromatin structure and has an important role in genome stability, DNA replication, and repair (Bayona‐Feliu et al., 2017; Fan et al., 2005; Hergeth & Schneider, 2015; Izzo et al., 2013).

Here we present that a de novo frameshift variant (c.416_419dupAGAA) within *HIST1H1E* in a patient with HIST1H1E syndrome. He had a severe ID and distinctive facial features including a high hairline, prominent forehead, and so on; most characteristics were prominently similar to previously reported in papers on patients with *HIST1H1E* variants (Burkardt et al., 2019; Duffney et al., 2018; Flex et al., 2019; Takenouchi et al., 2018; Tatton‐Brown et al., 2017). Given the molecular nature of the de novo frameshift variant (c.416_419dupAGAA) in *HIST1H1E*, it is strongly supported that this variant is pathogenicity. Interestingly, the variant of c.416_419dupAGAA in our patient and another 22 variants are reported in previous studies are all located in the C‐terminal domain, which suggests that the domain is a mutation “hot‐spot.” The C‐terminal domain of linker histones has a net positive charge, which is necessary to regulate higher order chromatin structure (Subirana, 1990; Tatton‐Brown et al., 2017). A net positive charge of the C‐terminus is necessary to binding linker DNA (Hendzel et al., 2004; Subirana, 1990; Tatton‐Brown et al., 2017). The truncated protein will likely impede the chromatin function and structure.

To explore the relationship between genotype and phenotype for *HIST1H1E* variants, we comprehensively curated variants‐related phenotypes. We identified some common phenotypes, such as craniofacial features, ID (DD), abnormal dentition, hypotonia, autism/behavior problem, brain MRI, ocular deformity, cardiac abnormalities, speech delay, motor delay, hands abnormalities, and RX abnormalities. We observed some shared clinical characteristics but also some diverse phenotypes. For instance, micrognathia, hypotonia, febrile seizures, downward slant palpebral fissures, deepset eyes, ptosis, prominent cheekbones have been observed in patient with the variant p.Thr142Lysfs*54 (Flex et al., 2019), but not observed in our patient with p.Ala141GlufsTer56. Contrarily, our patient had an upturned nasal tip, advanced bone age, atrial septum defect, bilateral, and hypothyroidism, whereas patient with the variant p.Thr142Lysfs*54 did not. Remarkably, although patients with the same variants, diverse phenotype was observed. For example, the frameshift variant (Ala144Glyfs*52) was identified in 14 patients. Co‐occurring manifestations, such as ocular deformity, speech delay, motor delay, scoliosis, skull abnormalities, and hands abnormalities were observed in all patients, but some different phenotypes were also identified in patients with the same variant, such as ID (13/14), hypotonia (12/13), autism/behavior problem (10/13), cardiac abnormalities (7/11), cryptorchidism (3/12), and several other features. Taken together, phenotypic heterogeneity indicates that it is a site‐specific effect on phenotypes for the variant occurring at different positions. A possible explanation of the diverse phenotype observed in patients with the same variant (Ala144Glyfs*52) is the difference in genetic backgrounds. However, the important phenotype of aging appearance as reported in previous studies was not observed in every subject included in this work, which suggests that aging appearance might represent a feature characterizing elderly.

The outstanding question that remains has not been able to answer in our current study is whether the underlying *HIST1H1E* gene variant determines the extent and severity of clinical features. Currently, because there are too few patients that have been determined, a strong genotype–phenotype analysis cannot be performed. Following the report of patients with the HIST1H1E syndrome is increased, which will be beneficial for a better understanding of genotype–phenotype correlations in the future.

In conclusion, the present findings facilitate current knowledge regarding the spectrum of *HIST1H1E* variants and related phenotypic features, which is helpful to assist diagnosis and clinical management of disease. The consistency and heterogeneity of phenotypes were observed in patients with different or same variants within *HIST1H1E*, which emphasizes the importantance of studying the site‐specific functions of variants. Our study will stimulate and facilitate research of *HIST1H1E* variants, which is important for the development of personalized treatments in the future.

## AUTHOR CONTRIBUTIONS

Clinical information collection: Wenjing Zhao and Yinhong Zhang. Data acquisition: Wenjing Zhao and Tao Lv. Data analysis/interpretation: Wenjing Zhao, Yinhong Zhang, Tao Lv, Jing He, and Baosheng Zhu. Manuscript preparation: Wenjing Zhao. Manuscript revision/review: Wenjing Zhao, Yinhong Zhang, Tao Lv, Jing He, and Baosheng Zhu. Manuscript final version approval: Wenjing Zhao and Baosheng Zhu.

## FUNDING INFORMATION

This work was supported by the National Natural Science Foundation of China (82160219), the Joint Special Research Funds of Kunming Medical University (202001AY070001‐156), the Special Foundation for Basic Research Program of Yunnan province (202101AT070233), the Doctor Foundation of the First People's Hospital of Yunnan province (KHBS‐2020‐018), the Talent Introduction Fund of the First People's Hospital of Yunnan province (KHYJ‐2019‐001), and Open Fund of Reproductive Obstetrics and Gynecology Clinical Center of Yunnan Province (zx2019‐01‐01, 2022LCZXKF‐SZ02).

## CONFLICT OF INTEREST STATEMENT

The authors declare no conflict of interest.

## ETHICAL APPROVAL

This study was approved by the Ethics Committee of First People's Hospital of Yunnan Province (Affiliated Hospital of Kunming University of Science and Technology). Written informed consent was signed by the individual's parent. Written informed consent was obtained from the family in the study.

## Supporting information


Table S1

## Data Availability

All data needed to evaluate the conclusions are presented in the article. The data that support the findings of this study are available from the corresponding author upon reasonable request.
